# Antioxidative Effect of Sage (*Salvia officinalis* L.) Macerate as “Green Extract” in Inhibiting the Oxidation of Fish Oil

**DOI:** 10.3390/antiox11010100

**Published:** 2021-12-30

**Authors:** Agnieszka M. Hrebień-Filisińska, Artur Bartkowiak

**Affiliations:** 1 Department of Fish, Plant and Gastronomy Technology, Faculty of Food Sciences and Fisheries, West Pomeranian University of Technology, 71-459 Szczecin, Poland; 2 Center of Bioimmobilisation and Innovative Packaging Materials, Faculty of Food Sciences and Fisheries, West Pomeranian University of Technology, 71-270 Szczecin, Poland; artur.bartkowiak@zut.edu.pl

**Keywords:** antioxidant, green extract, macerate, sage, fish oil

## Abstract

The aim of the study was to assess the antioxidant effect of concentrated oil macerate of sage (M) as a “green extract” in inhibiting the oxidation of Fish Oil (FO). In the homogenization-assisted maceration process, FO was used as a solvent for the sage active substances to produce M, which was then added to FO (25% *w*/*w*) and evaluated for its effect by monitoring the level of oxidation during refrigerated and room temperature storage. The macerate also examined polyphenols, plant pigments, DPPH antioxidant potential, oxidation level and sensory quality. It was shown that the maceration process made it possible to obtain aromatized M, containing polyphenols (carnosic acid, carnosol) and pigments, but with an increased level of peroxides, free fatty acids, compared to the control oil. M showed antioxidant properties and inhibited FO oxidation. It showed the best efficiency in FO during refrigerated storage, in the third month it reduced the level of peroxides by about 9 times, compared to the control. M retains unchanged quality at refrigerated temperature for up to 3 months. Sage macerates are “green extracts” that can be used as effective natural antioxidant additives, following preparation improvements to reduce the amount of peroxide formed.

## 1. Introduction

The sensitivity of edible oils to oxidative processes increases with the number of double bonds (unsaturated fatty acids) in the molecule. Therefore, it is known that fish oils are very susceptible to oxidation.They are a source of PUFA n-3 unsaturated polyene fatty acids, mainly EPA (eicosapentaenoic acid) and DHA (docosahexaenoic acid). Their beneficial effects on the human body are very well documented. However, oxidation processes can drastically reduce their nutritional and sensory quality and reduce the health-promoting effect on the body [1]. Inhibition of unfavorable oxidation processes is possible through the use of antioxidants. Among the antioxidants, synthetic and natural are distinguished. Synthetic antioxidant preparations may have an adverse effect on human health. Recently, natural food additives have been very popular [2,3].

The most common source of natural antioxidant compounds are plants. Plant-derived antioxidants are mainly polyphenols (phenolic acids, flavonoids, anthocyanins, lignans and stilbens), carotenoids (xanthophylls and carotenes) and vitamins (vitamins E and C) [4]. Extraction with organic solvents (e.g., maceration and Soxhlet extraction) or hot water bath is usually used to extract antioxidants from plant material. These methods are very time-consuming, require relatively large amounts of solvents at low extraction yields. In addition, a long extraction time may lead to the degradation of thermolabile compounds [4]. Another way to isolate antioxidants from plant tissues is the use of unconventional methods (e.g., supercritical fluid extraction, pressurized liquid extraction, high hydrostatic pressure extraction, pulsed electric field extraction). Unconventional methods usually allow to shorten the time and obtain better extraction efficiency, but are associated with high investment costs [4]. Maceration with edible oils can be an alternative method. This method belongs to the “green extraction methods” because it does not require the use of organic solvents and has no harmful effects on the environment and health. It is a natural, completely safe and simple method of extracting active ingredients from plant material using edible oils. It consists in combining edible oils, e.g., with herbs, which enables direct migration of active ingredients to the oil phase, and then the liquid-oil phase is separated from the solid phase [5]. There are no intermediate steps, and the obtained oil extract is distinguished by a low degree of processing. The macerate obtained in the maceration process is simply oil (depending on what was used), enriched with plant ingredients extracted from plants. The high concentration of bioactive ingredients in the macerate may allow it to be used as an additive to other oils or some food products. Food without artificial additives is often chosen by consumers. Therefore, research on the development of new possibilities of extending the shelf life of the food, based not only on natural ingredients, but also methods, has its strong justification.

Commonly, the maceration process is usually used to flavor edible oils with aromatic plants. This method enriches the oil with not only aromatic compounds but also antioxidants [6]. The maceration process can take place in a conventional manner, which consists in naturally soaking the plants in oil, usually for 14–15 days [7,8], but sometimes longer—45 days [9]. Supporting maceration with additional treatments allows you to significantly shorten the maceration time from several weeks to several or several minutes [6,10]. The maceration process can be supported by mixing [11], homogenization [5,12] and the use of ultrasound [6,10,13], or microwave heating [14]. Various herbs and spices (fresh and dried) are most often used to flavor edible oils [7,8,9,11]. So far, the studies have produced and tested macerates with rosemary, sage, summer savory, laurel, oregano, garlic, basil, thyme, lavender, menthe, chill pepper [6,7,8,9,10,11,12,13,14,15,16,17]. Among the plants used for maceration and aromatization of oils, rosemary has proven to be one of the most suitable herbs. It increased the antioxidant capacity and increased the oxidative stability of olive oil [6,7,15]. The most commonly used and best-studied oil for maceration is olive oil [6,7,8,9,10,11,12,13,14,15,16,17]. Other oils are used less frequently in studies: sunflower [12], oils from the seeds of black cumin, borage, evening primrose, safflower, walnut, common hazel, and oilseed rape, the flesh of sea-buckthorn fruits [16]. There is little data on the aromatization of fish oils. Only in the previous work, a concentrated macerate of cod liver oil and sage was obtained, which was then added to the oil in various amounts and its stability was tested in the accelerated oxidation test with UV rays [5]. However, there is no data on the effect of sage maceration on fish oil under natural conditions, when stored at refrigerated and room temperature. The results obtained in accelerated oxidation tests do not always coincide with the results obtained in natural conditions.

Naturally, fish oil is devoid of active plant ingredients, compared to vegetable oils, especially unrefined virgin oils, which can be a rich source of polyphenols, carotenoids, etc. Maceration of fish oil with sage can allow it to be enriched the oil with bioactive ingredients of sage, which can improve not only its durability, smell, taste, but also its pro-health value. Sage is known for its antioxidant, anti-inflammatory, antibacterial and anticancer properties [18]. It is also prescribed strong antiviral properties, also against SARS-CoV-2 [19,20], which in the current world situation is of great importance and responds to the consumer’s needs. Medicinal sage is very similar to rosemary and has similar bioactive ingredients to rosemary. Therefore, like rosemary, sage may prove to be a suitable species for maceration with edible oils. Primarily, carnosic acid and its derivatives are responsible for the antioxidant properties of both species. Among many polyphenols present in sage and rosemary, carnosic acid is characterized by increased solubility in lipids [5]. Therefore, during maceration, this compound can be extracted from the sage into the oil phase. The presence of polyphenols in the macerate may affect the the increase of antioxidant properties. In previous studies, oil macerates of sage inhibited the oxidation of cod liver oil during the accelerated oxidation test with UV rays [5]. Presumably, the macerate may also exhibit antioxidant activity during the storage of fish oil. The aim of the study was to assess the quality of a concentrated aromatized macerate obtained from sage and fish oil in the process of homogenization-assisted maceration as a “green” natural antioxidant in extending the shelf life of fish oil during storage at refrigerated temperature, in the dark (for 6 months) and at room temperature (for 3 weeks) with exposure to daylight.

## 2. Materials and Methods

### 2.1. Fish Oil

Fish oil (FO): cod liver oil (LYSI, Reykjavik, Iceland) was used, without the addition of antioxidants. The oil was stored in a refrigerator at 4 °C, in 24 kg barrels, after the barrel was opened and the oil portion was taken for analysis, the barrel was filled with nitrogen and sealed tightly.

### 2.2. Sage

The research was carried out on the medical sage (*Salvia officinalis* L.) variety “Bona” from an experimental farm in Szczecin (Poland) belonging to the Department of Horticulture of the West Pomeranian University of Technology in Szczecin. The sage herb was dried naturally after harvest (August) in dark conditions (temperature 20 to 23 °C). The dried leaves of plants were ground in a grinder to a powdered form, with a particle diameter of up to 0.4 mm, and then stored in sealed foil bags in a dark place until analysis, but not longer than four weeks.

### 2.3. Sage Macerate

The oil macerate of sage was obtained by mixture of ground sage with fish oil (weight ratio of sage to oil: 1 to 5.7), homogenization (1 min, rotational speed 10,000 rpm, knife homogenizer, POL-EKO, Wodzisław Śląski, Poland) and then filtration. After separating the plant particles, the macerate was used to stabilize the fish oil and analyzes were performed. The maceration process, procedure and conditions were described in the previous work [5].

### 2.4. Description of The Conducted Research

**Figure 1:**
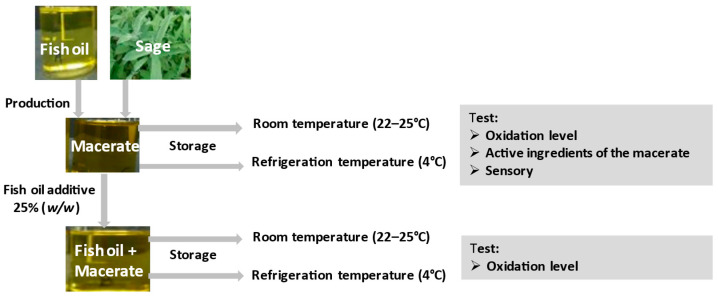
The scheme of the experiment.

The description of the experiment is schematically presented in Figure 1.

In order to test the antioxidant properties of macerate, fish oil with 25% addition of macerate was stored and native oil (as a reference sample) was stored in two variants: (1) at room temperature (22–25 °C) for 3 weeks, in glass, clear and closed bottles (250 cm^3^), in a place well-lit by daylight (the samples were tested on the day the experiment was set up and after two and three weeks of storage; (2) at refrigerated temperature (4–5 °C) for 6 months in the dark, in closed bottles, additionally wrapped with aluminum foil (trials were tested on the day the experiment was set up, after two weeks, and then after one, two, three and six months).

The concentration of macerate in fish oil and stabilization parameters were selected on the basis of previous studies [5]. The oxidation state was monitored during storage. Concentrated macerate (100%) was also tested, which was stored analogously in two temperature variants. Its oxidation level, total polyphenol content, carnosic acid and carnosol content, DPPH antioxidant activity and color were examined. In the sage used to obtain the macerate, the following were determined: total carotenoids, chlorophyll, total polyphenols and DPPH antioxidant activity.

### 2.5. Determination of the Oxidation Level

Peroxide Value (PV) according to norm [21] (measurement of primary oxidation products in meqO/kg of fat). The oil/macerate was dissolved in chloroform, acetic acid (15 cm^3^) and saturated potassium iodide were added. After 5 min, the iodine was titrated with 0.002 N sodium thiosulfate in the presence of a few drops of starch solution.

Anisidine Value (AV) according to norm [22] (measurement of secondary oxidation products). The oil was dissolved in chloroform followed by the addition of an anisidine reagent. After 10 min, the absorbance at λ = 350 nm was measured against a blank.

Totox index (measurement of the total content of oxidation products) calculated according to norm [22]:

Totox = 2 × PV + AV(1)

Acid Value (AcV) according to norm [23] in mg KOH/g fat. The content of free fatty acids was determined by the titration method. To 10 cm^3^ of the solution (oil dissolved in chloroform) were added 5 cm^3^ of methanol and 0.5 cm^3^ of phenolphthalein solution, then the samples were titrated with 0.01 mol/L KOH solution.

Iodine Value (IV) according to norm [24] in g I/100 g of fat (determines the content of unsaturated compounds, measurement of double bonds). Wijs reagent was added to the oil sample. After two hours, 10 cm^3^ of 10% potassium iodide and 75 cm^3^ of water were added and titrated with 0.1 mol/L sodium thiosulfate solution.

### 2.6. Extraction of Active Compounds (Polyphenols) from the Macerate to Hydrophilic Phase

The macerate was dissolved in hexane and shaken with 70% methanol, then the samples were extracted in an ultrasonic bath according to [5]. In the obtained extract, the antioxidant activity of DPPH, the total content of polyphenols and the identification of polyphenols using the chromatographic method (HPLC) were determined.

### 2.7. Determination in the Macerate

The Total Content of Polyphenols was determined according to Singleton and Rossi [25] by spectrophotometric method with the Folin-Ciocalteau reagent, against caffeic acid as a standard. Folin-Ciocalteau reagent (5 cm^3^) and saturated calcium carbonate (10 cm^3^) were added to the macerate extract (5 cm^3^), after making up to 100 cm^3^ with water and mixing the whole sample, set aside in the dark place. After 1 h, the absorbance of the blue solution was measured at λ = 760 nm. The results are presented in mg of polyphenolic compounds expressed as caffeic acid in 100 g of macerate.

The Antioxidant Activity of DPPH was determined according to Yen and Chen [26]. The principle is to colorimetrically measure the degree of DPPH (1,1-diphenyl-2-picrylhydrazyl) reduction in the extracts. DPPH solution was prepared by dissolving it in methanol. To 1 cm^3^ of the diluted extract (1:9) were added 3 cm^3^ of methanol and 1 cm^3^ of DPPH solution, and after mixing, the samples were left in the dark place. After 10 min, the absorbance of the samples and of the blank sample against methanol were measured at λ = 517 nm. The calculated percent inhibition of DPPH was calculated by the formula of Rossi et al. [27]:

% DPPH = 100 − (Ap/A0) × 100(2)

where: Ap—sample absorbance; A0—absorbance of the blank sample.

Color was determined according to norm [28], using the formula:

C = 1000 × (A442 + A668)(3)

where: A442—absorbance of 1 cm^3^ of macerate and 10 cm^3^ of hexane at λ = 442 nm (carotenoids); A668—absorbance of 3 cm^3^ of macerate and 3 cm^3^ of hexane at λ = 668 nm (chlorophyll).

Identification of phenolic compounds in the macerate was performed by liquid chromatography (HPLC). An Agilent 1260 Infinity II liquid chromatograph coupled with a PDA detector was used. The separation was carried out on a Nucleosil 120-5 C18 reverse phase column with dimensions 250 × 4.6 mm at ambient temperature. The mobile phase consisted of acetonitrile (solvent A) and water with 5% acetic acid (solvent B). The flow rate was kept at 0.5 mL/min. The gradient program was as follows: 15% A/85% B 0–12 min, then changed linearly to: 0% A/100% B at 30 min, followed by a change to 85% A and 15% B at 50 1 min, the 85% A and 15% B system was held for another 10 min. Total analysis time 60 min. The injection volume was 20 µL and the peaks were monitored at 280 and 325 nm. Macerate extracts were filtered through a 0.45 µm membrane filter prior to injection. Preparation of standards: carnosic acid, carnosol, rosmarinic acid, caffeic acid, dissolved in methanol, filtered through a 0.45 µm filter and immediately injected onto the HPLC column. Retention time of the determined compounds [minutes]: Carnosic acid: 24.9–25; carnosol—23.2–23.3 (280 nm).

### 2.8. Research in Sage

The dry weight was determined by the drying method by drying sage samples to a constant weight at 105 °C [29].

Total carotenoids were determined using the methodology of Lichtenthaler and Wellburn [30]. The method consisted in the extraction of sage with 80% acetone in an ultrasonic cleaner (Ultrasonic cleaner SB-3200DTD, China, parameters: frequency—40 kHz, power—180 W and temperature 20 °C) for 5 min. The absorbance of the centrifuged extract was then measured at three wavelengths, 441, 646 and 663 nm, respectively. The result was quantified in mg total carotenoids in kg DM. sage.

Chlorophyll was determined according to Lichtenthaler and Wellburn [30]. As in the case of carotenoids, the extraction of sage with 80% acetone was carried out in an ultrasonic washer. Then the absorbance of the centrifuged extract was measured at the wavelengths: 646 and 663 nm. The result is presented in mg of the sum of chlorophyll a and chlorophyll b in 100 g DM. sage.

The total content of polyphenols was determined according to Singleton and Rossi [25] analogically as in the case of the macerate. The polyphenols were extracted from the dry, ground material with 70% methanol (40 cm^3^) in a water bath under reflux for half an hour. After cooling, the samples were filtered and made up to 100 cm^3^ with 70% methanol, thus obtaining an extract. The antioxidant activity of DPPH was determined according to Yen and Chen [26], analogously to the maceration. Sage extracts were obtained by extracting dried sage with methanol.

### 2.9. Sensory Analysis

Sensory analysis of macerate was performed by a team of 5 persons moderately experienced in sensory evaluation. The participants were tested for sensory sensitivity in standards [31,32]. Before starting the analysis, the team was well instructed on the principles of the study. Two initial training sessions were conducted prior to the relevant tests. the profile method was used to evaluate the taste and smell of macerate according to the method described in the ISO standard procedure. The intensity of the individual characteristics in the profile method was evaluated on a 10-point scale (where 0 is undetectable and 10 is very noticeable).

### 2.10. Statistical Analysis

The nonparametric Kruskal-Wallis test was used to assess the significance of differences between the groups, which is the equivalent of one-factor analysis of variance (ANOVA). The Mann-Whitney U test, which is the equivalent of the Student’s *t*-test, was used to assess the significance of differences between the two groups. The significance of the analyzed values was assumed for *p* < 0.05. The results were statistically elaborated using the STATISTICA 9.0 program.

## 3. Results

### 3.1. Characteristics of Sage

The composition of selected ingredients in the sage used to prepare the macerate is presented in Table 1.

### 3.2. Characteristics of Sage Macerate after Production

**Figure 2:**
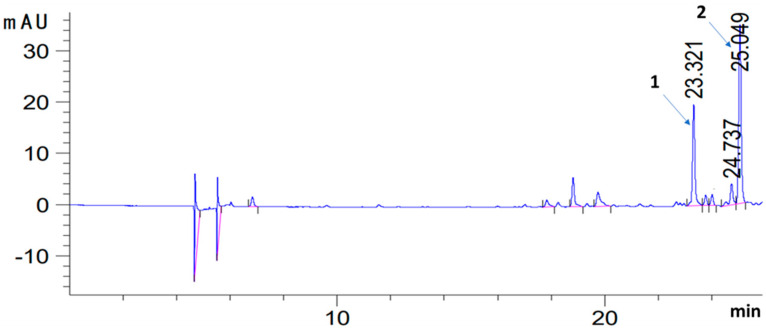
Chromatogram (HPLC) of an extract (70% methanol) from the sage macerate (1-carnosol, retention time—23.321 min; 2-carnosic acid, retention time—25.049).

Selected sage ingredients were extracted into the oil phase during maceration. The plant components were marked in the macerate: pigments and polyphenols, which influenced the antioxidant properties of the macerate, which is illustrated by the DPPH test result. On the basis of the chromatographic examination, carnosic acid and carnosol were identified in the macerate (Table 2, Figure 2).

The macerate preparation process increased the oxidation level. The macerate was characterized by an increased PV and AcV in relation to the native oil—used to prepare the macerate (Table 2). However, AV of the maceration was not higher than AV for the native FO. The level of the IV of the macerate after preparation was not lower than in FO, which was used to prepare the macerate.

**Figure 3:**
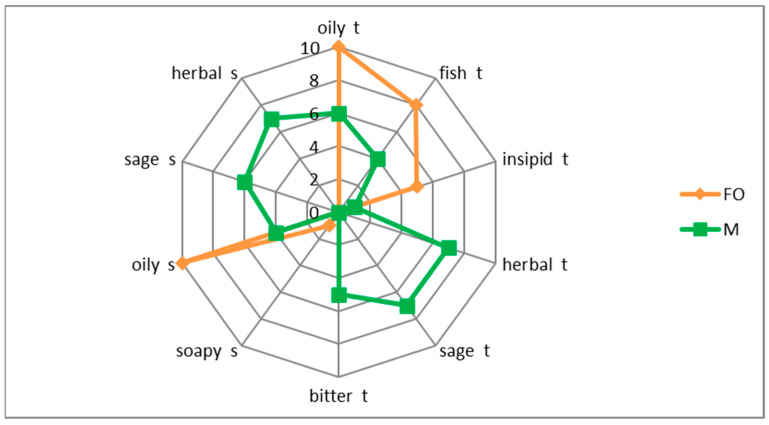
Taste (t) and odor (s) profile macerate (M, green) compared to pure cod liver oil (FO, orange).

The maceration process influenced the aromatization of the fish oil (Figure 3). Compared to the pure oil, the macerate had a herbal and sage aroma and flavor. There was also a bitter taste. The macerate also had a less appreciable aftertaste of fish oil.

### 3.3. Storage of Sage Macerate at Refrigeration Temperature j (4–5 °C) and Room (20–25 °C)

Along with the storage time of the macerate, both at room temperature and refrigerated temperature, PV, AV, AcV, Totox index increased, but at 4 °C the changes of these parameters were much slower (Table 3 and Table 4). However, a significant increase of PV, AcV, Totox only occurred at the end of the storage period, i.e., after 3 weeks—at room temperature (Table 3) and 6 months -at refrigerated temperature (Table 4). In the case of storage in cold stores after 1 month, a slight increase in the level of primary oxidation products (PV) was also observed. Similarly, the AV increased significantly after 5 and 6 months at refrigeration temperature (Table 4) and after 2 weeks in the macerate stored at room temperature (Table 3). Totox is a measure of the total amount of oxidation products and reflects the levels of both primary and secondary oxidation products. The increase of the AcV correlates with the increase in the level of free carboxylic acids, which most often may come from the hydrolysis of lipid molecules and indicate the detachment of fatty acids from glycerol in the lipid molecule.

The iodine value (IV) of the macerate during storage in most cases in both temperature variants was not lower than the iodine number of the fish oil used to prepare the macerate. Therefore, based on the data, it can be assumed that the oxidative changes of the macerate were not very deep, as there was no degradation of double bonds.

During the storage of the macerate, changes in the content of polyphenols, DPPH antioxidant activity and color were also observed (Table 5 and Table 6). At room temperature (in a lit place), along with the extension of the storage time, there was a clear decrease in the content of polyphenols, DPPH antioxidant activity and color (Table 5). However, at the refrigeration temperature, it was only in the sixth month of storage that the values of these parameters dropped significantly (Table 6). In the macerate stored under refrigerated conditions in the dark, the content of polyphenols, color and DPPH were very stable and did not change during the first 3 months of storage. In turn, in the macerate stored in a place illuminated by sunlight, at room temperature, there was a significant decrease in polyphenols, color and DPPH antioxidant activity after 3 weeks of storage. Chromatographic studies showed that the content of carnosic acid and carnosol changed during macerate storage, but the changes were not statistically significant (Table 7). In the case of carnosic acid, a clear decrease was noted after 6 months of storage. The content of carnosol decreased after 1 month of storage, and during the following months of storage, the level of carnosol remained at a similar level and did not change.

**Figure 4:**
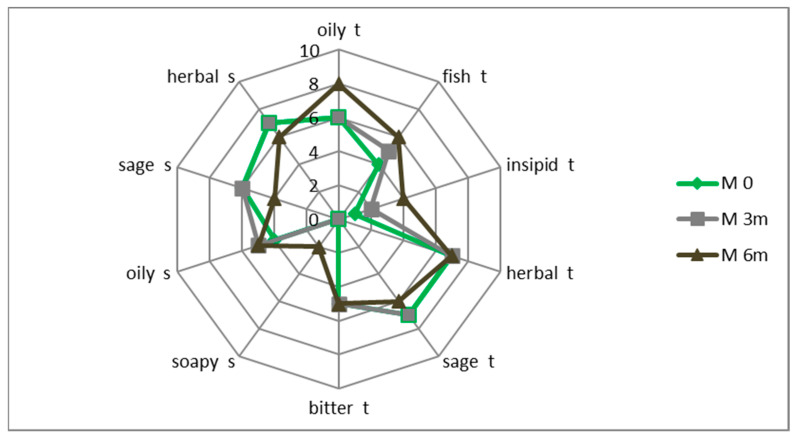
Macerate taste (t) and smell (s) profile after 3 (M 3 m) and 6 months (M 6 m) of refrigerated storage compared to the initial macerate (M 0).

After 3 months of storing the macerate at chilled temperature, its flavor and aroma profile did not change significantly compared to the original macerate (storage time 0). However, after 6 months, the changes were more intense. Fish, oily and soapy flavors were more pronounced, and sage and herb flavors were less noticeable (Figure 4).

### 3.4. Storage of Fish Oil Stabilized with Sage Macerate at Refrigeration Temperature j (4–5 °C) and Room (20–25 °C)

In order to test the antioxidant effect of the sage macerate—fish oil with 25% macerate (M/FO) was stored at room temperature (up to 3 weeks) and at refrigeration temperature (up to 6 months). During storage, the oxidation level of the samples was monitored compared to pure oil without the addition of the so-called reference trials (FO).

**Figure 5:**
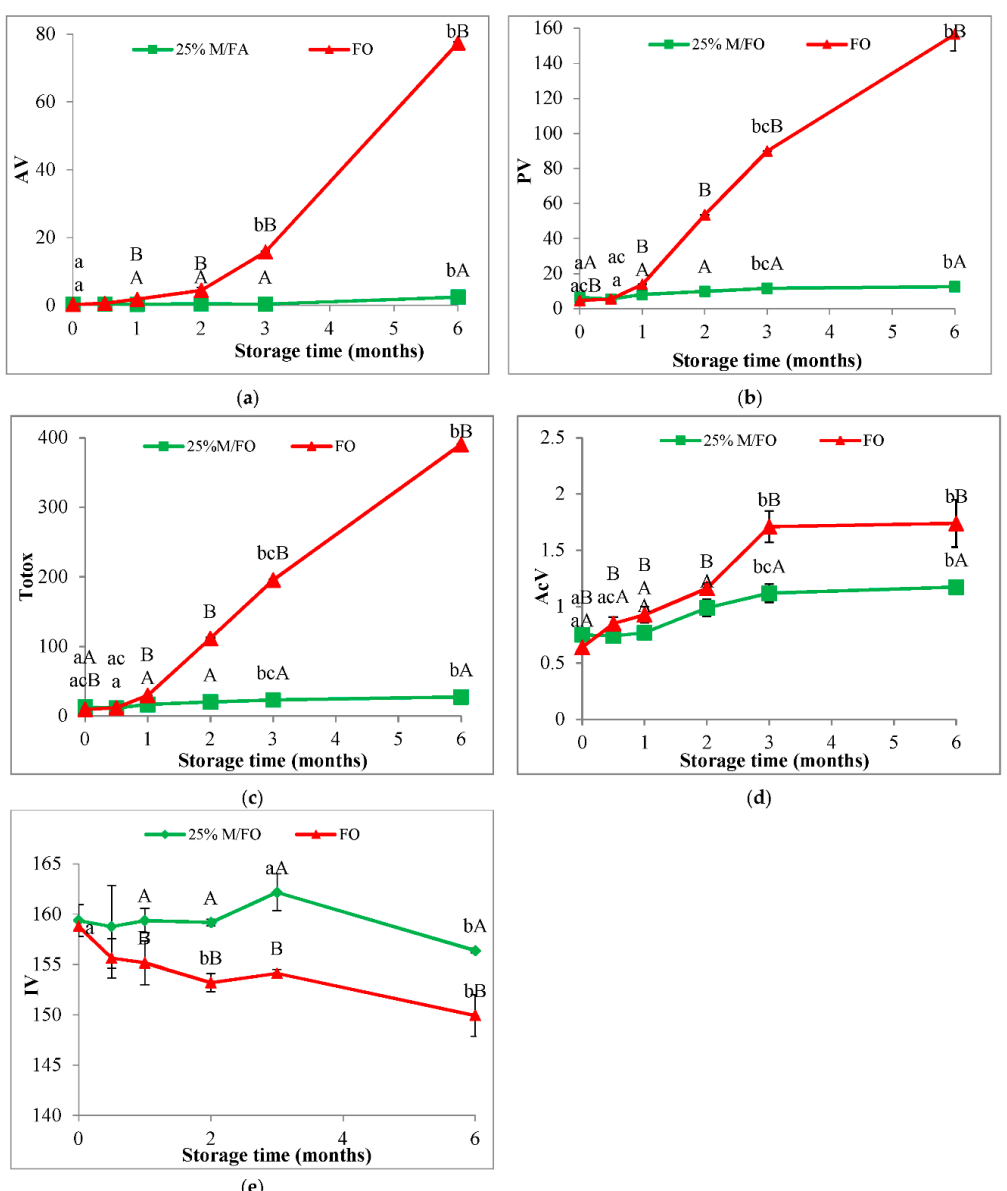
Anisidine value (**a**), peroxide value (**b**), Totox (**c**), acid value (**d**), iodine value (**e**) fish oil with macerate (25% M/FO, green) compared to control fish oil (FO, red) stored at refrigeration temperature (4 °C). (Values marked with the same lowercase letters a, b differ statistically significantly within the stored samples (25 M/FO or FO), while values marked with different uppercase letters—A, B differ significantly within the same storage time. Analogically values marked with the same letters do not differ statistically significantly, *p* < 0.05).

**Figure 6:**
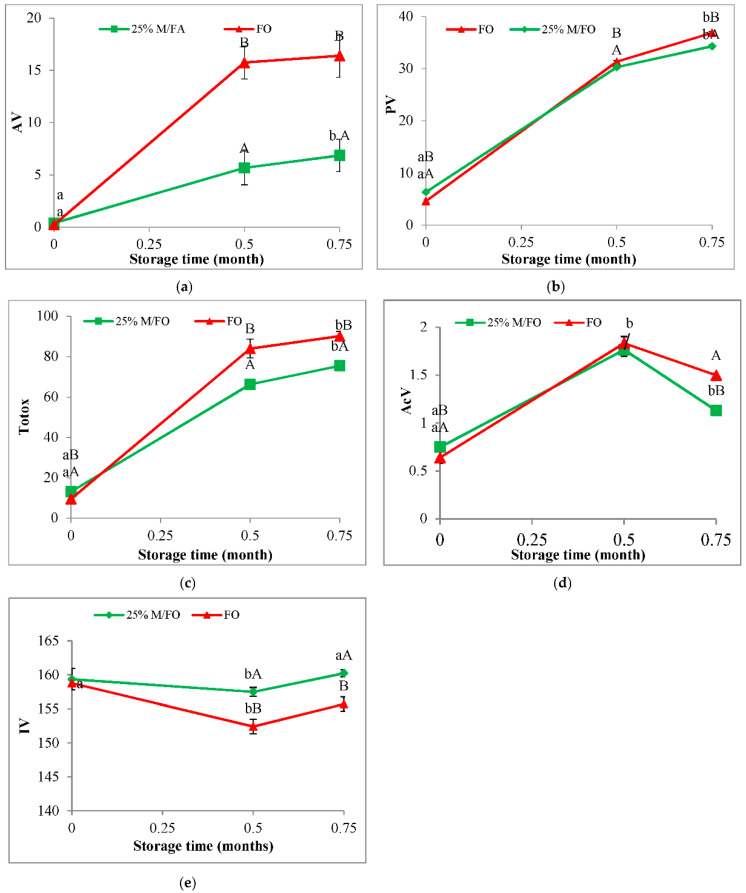
Anisidine value (**a**), peroxide value (**b**), Totox (**c**), acid value (**d**), iodine value (**e**) fish oil with macerate (25% M/FO, green) compared to control fish oil (FO, red) stored at room temperature (20–25 °C). (Values marked with the same lowercase letters a, b differ statistically significantly within the stored samples (25 M/FO or FO), while values marked with different uppercase letters—A, B differ significantly within the same storage time. Analogically values marked with the same letters do not differ statistically significantly, *p* < 0.05).

Studies have shown that sage oil macerate inhibits the oxidation of fish oil during refrigerated storage (Figure 5) and room temperature storage (Figure 6), but the fish oil retains better quality longer when refrigerated. At the beginning of the refrigeration storage period at 4 °C (time 0), an increase in the of PV, AV, AcV, Totox in the oil after adding the macerate (M/FO) was observed in relation to the control fish oil without the addition (FO) (Figure 5). However, after half a month of storage, there was a significant increase in oxidation in the control oil (FO) and an increase in deterioration indicators in relation to the macerate stabilized fish oil (M/FO). This tendency continued until the end of the storage period. It was observed that with the extension of the storage time in FO, the oxidation processes took place very quickly, while in M/FO there was a slower and slight increase in the concentration of oxidation products.

It was similar during storage of M/FO at room temperature (Figure 6). It was shown that the addition of macerate to fish oil significantly decreased the PV, AV, AcV, Totox in relation to the control oil without additives (FO), after 2 and 3 weeks of storage, respectively. In this case, however, the rate of oxidative changes was very fast and despite the antioxidant effect of macerate, it quickly achieved a high level of oxidation. On the other hand, at the refrigeration temperature, fish oil stabilized with sage macerate (M/FO) showed high stability compared to the control (FO). After two months, the PV of the oil stabilized with macerate (M/FO) was about five times lower compared to the value for the non-stabilized system (FO), and after 3 months it was almost 9 times lower. A similarly lower (4-fold) content of secondary oxidation products (AV) was determined in the M/FO after 3 months as compared to the control oil FO. Oil stabilized with macerate (M/FO) showed a very slow accumulation of peroxides during the entire storage period, while the level of secondary oxidation products did not change during the first three months.

The addition of macerate also protected against the degradation of double bonds in polyene fatty acids, as evidenced by the iodine value (IV), which in the case of the fish oil without macerate was significantly lower compared to the oil with macerate for most of the storage period at room temperature (Figure 6e) and refrigeration temperature (Figure 5e).

## 4. Discussion

During homogenization-assisted maceration, polyphenols, carotenoids and chlorophylls were extracted from the sage into the oil phase, hence their presence in the macerate. The content of carotenoids and chlorophyll in the macerate is evidenced by the value of the Color parameter, which in the case of the macerate is over twenty times higher than for the oil alone (FO). The Color (C) parameter is calculated according to the formula 3, which shows the close dependence of the Color (C) value on the concentration of carotenoids and chlorophyll in the determined system. The extracted compounds present in the macerate, in turn, influenced its antioxidant properties. Among the pigments in the macerate, carotenoids and chlorophylls may exhibit antioxidant properties, depending on environmental conditions [33,34]. The polyphenols [33] are known for their strong antioxidant properties, and they had a great influence on the ability to scavenge free radicals in the macerate in the DPPH test. The polyphenols in sage are characterized by great diversity in terms of polarity and solubility. Carnosic acid is at the lipophilic end of the scale, while rosmarinic acid is at the hydrophilic end [35].Chromatographic tests showed that carnosic acid and carnosol were extracted into the macerate. However, rosmarinic acid was not determined. The presence of carnosic acid and its derivatives (carnosol) is associated with its increased solubility in lipids. Whereas the extraction of rosmarinic acid into the oil phase was hampered by the hydrophilic properties of this acid. Thus, it can be concluded that the use of the oil to extract the antioxidant components from the sage is effective. The selection of an appropriate solvent for the extraction of bio-components from natural sources is of key importance for the efficiency of extraction, which in turn affects the antioxidant activity. For example, in the extraction of the bioactive 3-methoxy-4-hydroxycinnamic acid (PF5) fraction from the fungus (*Pleurotus florida*), methanol was most effective, while ethanol, water, ethyl acetate and hexane were not as effective as methanol [36].

Sensory tests have shown that aromatic compounds have also been extracted into the macerate. The macerate was characterized by a sage and herbal aroma and taste. Sage belongs to plants rich in essential oils which, due to their (lipophilic) properties, could have been extracted into the oil phase during the maceration process. The bitter taste in the macerate may also be related to the presence of essential oils and polyphenolic compounds. The flavoring ingredients of the sage partially masked the taste and aroma of fish oil (fish, oily). Similarly, in other studies [6,10] the maceration process was used to aromatize extra virgin olive oil with sage and rosemary. The obtained macerates were characterized by a high content of polyphenols, carotenoids and chlorophyll [10]. The authors used ultrasound in their research, which allowed to shorten the maceration time, compared to (conventional) maceration without support. In this study, maceration was supported with homogenization. Previous studies [5] showed that the use of homogenization for maceration of sage with cod liver oil allows to obtain the oil macerates enriched with polyphenols, plant pigments, characterized by the ability to capture free radicals in the DPPH test. Storage of the homogenized system (cod liver oil + sage) for another 2 weeks did not increase the antioxidant activity of DPPH in the macerate. On the other hand, it contributed to an intensive increase, by over 72%, of plant pigments and, to a small extent, of polyphenols (by over 13%). This additional portion of polyphenolic components and plant dyes extracted after 15 days of storage of the homogenized system (cod liver oil + sage) not only did not increase the DPPH antioxidant activity in the macerate, but also did not improve the effectiveness This additional portion of polyphenolic components and plant dyes, extracted after 15 days of storage of the homogenized system (cod liver oil + sage) not only did not increase the DPPH antioxidant activity in the macerate, but also did not improve the effectiveness in inhibiting fish oil oxidation during the UV test [5]. Therefore, it can be assumed that the antioxidant properties of the macerate were mainly influenced by the polyphenolic compounds of the macerate, derived from sage.

The process of preparing the macerate increased the oxidation level. After the maceration process, the amount of peroxides (PV) and free fatty acids (AcV) increased compared to the control oil (FO). An increase in the AcV may indicate hydrolytic changes in macerates towards the release of fatty acids from the fat molecule, or the breakdown of the molecule into glycerol and free fatty acids. The biochemical hydrolysis may have been caused by the activity of lipases. The source of these lipases in the maceration could be microorganisms that, along with the sage, could have entered the macerate. This was probably the cause of the partial degradation of the triacylglycerols, therefore the free fatty acids increased the acid number (AcV) level. It can also be assumed that the increase in the AcV in the macerate after preparation is related to the presence of polyphenols, and more specifically polyphenolic acids, which were extracted from the sage into the oil phase during maceration. The oxidative changes of the macerate, however, were not very advanced. This is evidenced by the result of anisidine value (AV) and iodine value (IV). The level of iodine number (IV) in the macerate after preparation was not lower than that of the fish oil that was used to prepare the macerate, while the level of anisidine value, i.e., the amount of secondary oxidation products, did not increase after maceration. The iodine value is a measure of the amount of unsaturated bonds, so it can be assumed that there was no degradation of unsaturated compounds. The homogenization used during the maceration process could cause aeration of the macerate and could contribute to the generation of primary oxidation products. Therefore, there was an increase in the PV value of the macerate compared to the PV of the fish oil used in the preparation of the macerate. Fish oil is very susceptible to oxidation due to the presence of polyene fatty acids, including EPA and DHA, which have double bonds [1]. EPA consists of 20 carbon atoms and has 5 unsaturated fatty acids, while DHA has 6 such bonds between 22 carbon atoms. For this reason, these acids: EPA and DHA are very exposed to oxidative processes. They oxidize quickly, and increased temperature, light and oxygen access accelerate these processes [1].

Similarly, in the previous work [5], an increase in the level of primary oxidation products (PV) was noted after macerate preparation (after homogenization), with no increase in the AV. Also in the studies by Soares et al. [6], after the preparation of macerates of rosemary and basil based on olive oil, there was a decrease in quality parameters: an increase in the acid value and a slight increase in the peroxide value, especially in the case of conventional maceration. The oxidative changes initiated at the macerate preparation stage were further deepened during macerate storage, and the pace of these changes depended on the storage conditions. Room temperature, light contributed to an increase in the rate of oxidation. Hence, in the case of the macerate stored at room temperature, the oxidation indexes (AV, PV, AcV, Totox) after about 3 weeks were higher compared to the oxidation level of the macerate stored in a cold store and protected from light after 6 months. Under refrigeration conditions (temperature 4 °C, in the absence of light), the macerate stored very well, its quality did not deteriorate for the first 3 months. Similarly, with regard to the content of polyphenols and the antioxidant activity of DPPH, the values of these indices did not change also during the first 3 months of refrigerated storage. The increase in oxidation and decrease in the content of polyphenols and DPPH activity were noted only after 6 months. On the other hand, at room temperature with sunlight, after 3 weeks, there was a clear decrease in the content of polyphenols, DPPH antioxidant activity and color, compared to the state at the beginning of storage (time 0), which was probably related to sunlight and increased temperature, i.e., accelerating oxidation processes, not only of fatty acids but also of polyphenols. This situation is related to the level of carnosic acid retention. During the storage of the macerate at refrigeration temperature, there was a decrease in carnosic acid after 6 months of storage. Therefore, a decrease in the total content of polyphenols and DPPH activity was noted. Carnosic acid is considered unstable. However, it is more stable in fats [5] and fish oils than in polar solvents [37].

After production, macerate was added to the cod liver oil in an amount of 25% *w*/*w*. The system was then stored in two variants: at room temperature (22–25 °C, with access to light) for 3 weeks and at cooling temperature (4–5 °C, without light) for up to 6 months to test the antioxidant effect. The amount of macerate addition at the level of 25% was selected on the basis of previous studies [5]. The studies showed that the macerate inhibited the oxidation of the oil at refrigerated and room temperature, but the fish oil was more stable under refrigeration conditions. The obtained sage macerate was characterized by antioxidant properties in the DPPH test, therefore, when added to fish oil, it inhibited the oxidation processes during its storage. The antioxidant effect of macerate, as already mentioned, results from its content mainly of polyphenols. Polyphenols are characterized by a very strong antioxidant capacity, which results from their ability to scavenge free radicals, donate hydrogen atoms, electrons and chelate metal cations (oxidation catalysts) [33]. Sage is very rich in a variety of polyphenolic compounds with different properties. It contains both polar and lipophilic polyphenols [35]. The highest antioxidant properties among sage polyphenols are mainly prescribed for carnosic acid, carnosol and rosmarinic acid [38]. Among the polyphenols of sage, carnosic acid has the most lyophilic properties [35], therefore chromatographic studies have confirmed its presence in the macerate. The strong properties of carnosic acid and its derivatives result from the presence of two ortho hydroxyl groups on the C_11_ and C_12_ carbon. Carnosic acid can also act as a “cascade” antioxidant in which subsequent products of the oxidation reaction still perform an antioxidant function [3]. Carotenoids and chlorophylls also show antioxidant properties [34]. However, chlorophylls in the presence of light can accelerate oxidative processes [39], therefore, when storing fish oil with macerate in conditions with access to light and higher (room) temperature, chlorophylls could act as oxidation catalysts. However, in the dark, chlorophylls may show some antioxidant activity. Recently, Pérez-Gálvez et al. [34] devote a lot of space in their work to the antioxidant property of chlorophyll. In fish oil, after the addition of macerate, an increase in AcV, PV was observed at the beginning of the storage period. The macerate itself already contained a certain portion of free acids and peroxides, therefore, after adding to the oil, AcV, and PV increased. As the storage time was extended, the rate of oxidative changes in pure oil was very fast, while in the oil with macerate there was a slower and slight increase in the concentration of oxidation products.

At the beginning, during the first month of storing the control oil, there was a slow accumulation of peroxides, which is characteristic of the induction period, and then there was a sharp increase in their amount, characteristic for the propagation phase. During oxidation, oxygen reacts with unsaturated fatty acid bonds to form peroxides, i.e., primary oxidation products, which can be determined by the peroxide number. Over time, the formed peroxides undergo further transformations and the formation of aldehydes and ketones, referred to as secondary oxidation products [39], therefore an increase in the anisidine number is observed. In this experiment, especially after the 3-month storage of the oil (at refrigerated temperature) without the addition of antioxidant, quite intensive accumulation of secondary products was observed.

The macerate showed antioxidant activity and inhibited the oil oxidation. Maceration with oils enables the extraction of antioxidants from sage and is a good method of obtaining oil preparations rich in carnosolic acid as antioxidant additives for a wide range of products.

## 5. Conclusions

Macerate obtained from fish oil and sage is characterized by the presence of polyphenols, plant pigments, and shows DPPH antioxidant activity. A high level of carnosic acid with special antioxidant properties was determined in the macerate. When added to the oil in the amount of 25% (*w*/*w*), it significantly slowed down the formation of oxidation products, compared to the control fish oil itself (without additives) during the storage of the oil at refrigerated temperature and at room temperature. Despite the unfavorable changes (an increase in the peroxide value and the acid value after homogenization), which caused a decrease in the quality of the macerate already during its preparation, the macerate, however, after adding it to the oil, showed an antioxidant effect and extended its shelf life. At room temperature, the rate of oxidative changes was very fast and despite the antioxidant effect of the fish oil macerate in a short time (after half a month), it reached a high level of oxidation significantly exceeding the adopted limits. On the other hand, at 4 °C, the oil stabilized with sage macerate showed high stability compared to the non-stabilized fish oil, especially for the first 3 months.

Macerate is a safe, natural “green extract” that can be used not only as an antioxidant for oils, but also for other food products. However, research on the improvement of the macerate should be continued so that the production process does not increase the oxidation level. Considering the pro-health and multidirectional effects of fish oil and sage, the macerate may also become a functional preparation or a supplement with beneficial pro-health effects on the human body, including a treatment aid and preventive agent against SARS-CoV-2.

## Figures and Tables

**Table 1:** Content of selected ingredients in sage.

Dry Matter (%)	Carotenoids (mg/kg d.m.)	Chlorophyll (mg/100 g d.m.)	Polyphenols (mg/100 g d.m.)	DPPH (%)
92.1 ± 0.12	997 ± 0.78	633 ± 9.75	11,177 ± 47	27.9 ± 0.49

**Table 2:** Parameters of the sage macerate and fish oil used to obtain the macerate.

Tested Parameter	Macerate	Fish Oil
Carnosic acid (mg/g)	1.116 ± 0.034	-
Carnosol (mg/g)	0.376 ± 0.020	-
Polyphenols (mg/100 g)	177.5 ± 2.13	-
DPPH (%)	31.0 ± 2.86	-
Color	1443 ± 18.38 ^B^	61 ± 3 ^A^
PV (meqO/kg)	13.0 ± 0.11 ^B^	4.63 ± 0.24 ^A^
AV	0.11 ± 0.08 ^B^	0.23 ± 0.08 ^A^
Totox	26.1 ± 0.14 ^B^	9.5 ± 0.34 ^A^
AcV (mg KOH/g)	1.00 ± 0.14 ^B^	0.64 ± 0.06 ^A^
IV	162.5 ± 1.10 ^B^	158.8 ± 0.51 ^A^

^A,B^ U-Mann-Whitney test—values marked by letter symbols: A, B differ significantly.

**Table 3:** Anisidine value (AV), peroxide value (PV) Totox, acid value (AcV) and iodine value (IV) of sage macerate after various storage times at room temperature (22–25 °C).

Storage Time (Months)	AV	PV (meqO/kg)	Totox	AcV (mg KOH/g)	IV
0	011 ± 0.08 ^a^	13.0 ± 0.11 ^a^	26.1 ± 0.14 ^a^	1.00 ± 0.14 ^a^	162.5 ± 1.10 ^a^
0.5	3.07 ± 1.97 ^b^	29.6 ± 0.24	61.6 ± 3.43	2.06 ± 0.08	158.5 ± 1.56 ^b^
0.75	1.95 ± 0.79	33.1 ± 0.77 ^b^	68.1 ± 0.76 ^b^	2.2 ± 0.08 ^b^	159.0 ± 2.14

^a,b^ Kruskal-Wallis test—values read in separate columns marked with different letters a, b differ significantly from each other, marked with the same letters a, a or b, b do not differ significantly from each other, *p* < 0.05.

**Table 4:** Anisidine value (AV), peroxide value (PV) Totox, acid value (AcV) and iodine value (IV) of sage macerate after various storage times at refrigeration temperature (4 °C).

Storage Time (Months)	AV	PV (meqO/kg)	Totox	AcV (mg KOH/g)	IV
0	0.11 ± 0.08 ^a^	13.0 ± 0.11	26.1 ± 0.14	1.00 ± 0.14 ^a^	162.5 ± 1.10 ^a^
0.5	0.51 ± 0.18	9.57 ± 0.28 ^a^	19.7 ± 0.74 ^a^	1.14 ± 002 ^a^	156.1 ± 2.36 ^b^
1	0.7 ± 0.17	13.3 ± 0.04 ^b^	27.3 ± 0.36 ^b^	1.20 ± 0.06	157.7 ± 0.19
2	0.7 ± 0.16	12.8 ± 0.19	26.2 ± 0.21	1.28 ± 0.1	160.5 ± 3.05
3	1.3 ± 0.08 ^b^	12.54 ± 0.36	26.4 ± 0.64	1.32 ± 0.14	159.8 ± 0.17
6	2.46 ± 1.09 ^b^	15.4 ± 0.21 ^b^	33.2 ± 0.67 ^b^	1.55 ± 0.05 ^b^	158.0 ± 1.73

^a,b^ Kruskal-Wallis test—values read in separate columns marked with different letters a, b differ significantly from each other, marked with the same letters a, a or b, b do not differ significantly from each other, *p* < 0.05.

**Table 5:** Polyphenol content, DPPH activity, color of macerate depending on the storage time at room temperature (22–25 °C).

Storage Time (Months)	Polyphenol (mg/100 g)	DPPH (%)	Color
0	177.5 ± 2.13 ^a^	31.0 ± 2.86 ^a^	1443 ± 18.38 ^a^
0.5	139.1 ± 9.71	11.7 ± 1.44	1030,5 ± 12.02
0.75	123.9 ± 6.38 ^b^	8.14 ± 032 ^b^	979 ± 15.56 ^b^

^a,b^ Kruskal-Wallis test—values read in separate columns marked with different letters a, b differ significantly from each other, marked with the same letters a, a or b, b do not differ significantly from each other, *p* < 0.05.

**Table 6:** Polyphenol content, DPPH activity, color of macerate depending on the storage time at refrigeration temperature (4 °C).

Storage Time (Months)	Polyphenol (mg/100 g)	DPPH (%)	Color
0	177.5 ± 2.13	31.0 ± 2.86	1443 ± 18.38 ^a^
0.5	182.1 ± 1.72	29.3 ± 1.11	1331 ± 13.44
2	181.8 ± 2.32	31.5 ± 0.48 ^a^	1391 ± 28.99
3	188.5 ± 5.04 ^a^	31.1 ± 0.48	1307 ± 21.92 ^b^
6	151.5 ± 0.61 ^b^	26.7 ± 1.02 ^b^	−

^a,b^ Kruskal-Wallis test—values read in separate columns marked with different letters a, b differ significantly from each other, marked with the same letters a, a or b, b do not differ significantly from each other, *p* < 0.05.

**Table 7:** The content of carnosic acid and carnosol in macerate depends on the storage time at refrigerated temperature (4 °C).

Storage Time (Months)	Carnosic Acid (mg/g)	Carnosol (mg/g)
0	1.116 ± 0.034	0.376 ± 0.020
0.5	1.062 ± 0.035	0.318 ± 0.020
1	1.226 ± 0.035	0.194 ± 0.005
2	1.117 ± 0.048	0.218 ± 0.030
3	1.130 ± 0.083	0.200 ± 0.007
6	0.873 ± 0.060	0.209 ± 0.010

## Data Availability

The data used to support the findings of this study are available from the corresponding author upon request.
